# Combinations of compound cold medicines should be used with caution: a case series

**DOI:** 10.3389/fmed.2025.1513019

**Published:** 2025-03-13

**Authors:** Jinlin Guo, Tianning Zhang, Shaohui Song, Junwei Li

**Affiliations:** ^1^Department of Pharmacy, Shanxi Provincial People’s Hospital, Taiyuan, Shanxi, China; ^2^Department of Pharmacy, Yunnan Infectious Diseases Hospital, Kunming, Yunnan, China; ^3^Department of Pharmacy, The Maternal and Child Health Hospital of Dadukou District of Chongqing City, Chongqing, China; ^4^Department of Neurosurgery, Shanxi Provincial People’s Hospital, Taiyuan, Shanxi, China

**Keywords:** common cold, compound cold medicines, chronic disease, drug–drug interactions, adverse reactions

## Abstract

**Background:**

Compound cold medicines contain multiple ingredients and are widely used to alleviate discomfort caused by viral infections. It is generally believed that compound cold medicines rarely cause serious adverse reactions; therefore, patients do not need a prescription for them. Many individuals suffer from chronic illnesses and must take medications for prolonged periods. These patients may experience serious drug–drug interactions if they self-administer compound cold medicines. Here, we report three cases of severe adverse drug reactions induced by compound cold medicines.

**Case presentation:**

All patients had a chronic disease and had been taking medication for a long time without experiencing serious adverse reactions. However, after self-administering a compound cold medicine, serious drug–drug interactions occurred. In Case 1, a 67-year-old woman with no history of coronary artery disease or angina developed angina after concurrently taking diltiazem and compound methoxyphenamine. In Case 2, a 65-year-old man who was taking propylthiouracil for a year without any adverse reactions experienced mental status abnormalities and acute liver failure after taking “CONTAC NT.” In Case 3, a 63-year-old man, who was taking levodopa, entacapone, and selegiline for a long time, without any apparent adverse reactions, developed serotonin syndrome after adding CONTAC NT. These issues were resolved after the discontinuation of medication.

**Conclusion:**

Patients should consult a pharmacist or physician before using compound cold medicines to avoid the risk of adverse reactions caused by drug–drug interactions.

## Background

The “common cold” is defined as an upper respiratory viral infection; symptoms often include fever, headache, muscle aches, runny nose, and cough (1). The common cold is a benign, self-limiting illness that typically resolves with symptomatic treatment and does not require antibiotics. Compound cold medicines, such as “CONTAC NT” (Sino-American Tianjin SmithKline and French Lab., Ltd., Tianjin, China), contain multiple ingredients including paracetamol (500 mg), chlorpheniramine maleate (2 mg), dextromethorphan (15 mg), and pseudoephedrine (30 mg) in each tablet, which can alleviate discomfort caused by upper respiratory infections. In China, a vast majority of compound cold medicines are available over-the-counter for easy public access. Elderly individuals often suffer from conditions, such as hypertension and diabetes, which require long-term medication. The concurrent use of multiple medications by patients with chronic diseases following the development of the common cold may increase the risk of drug–drug interactions. Herein, we report three cases of severe adverse drug reactions caused by compound cold medicines.

## Case presentation

### Case 1

A 67-year-old woman (the first author’s mother), who no history of coronary heart disease and had not experienced angina pectoris in the past 12 months, had been taking diltiazem (15 mg twice daily) for paroxysmal supraventricular tachycardia for many years. The patient’s blood pressure was monitored daily, with values of 90–100/55-68 mmHg, and a heart rate of 53-65/min. She underwent check-up 1–2 times a year, with the most recent visit 5 months before presentation. At this point, the results of the physical examination showed that she had normal blood glucose, a triglyceride level of 1.93 mmol/L, and premature atrial contractions. She experienced cough due to coronavirus disease (COVID-19) infection and self-administered compound methoxyphenamine capsules (methoxyphenamine hydrochloride: 12.5 mg, noscapine: 7 mg, aminophylline: 25 mg, chlorpheniramine maleate: 2 mg) at home on the evening of February 17, 2024. Thirty minutes after taking the medication, she developed squeezing chest pain in the precordial area, with a heart rate of 112 beats/min and normal blood pressure. She was suspected of experiencing an angina attack, and was immediately administered nitroglycerin tablets (0.5 mg) and metoprolol, with nitroglycerin administered every 10 min, three times in total, to relieve the angina. The following afternoon, she took compound methoxyphenamine capsules again, and angina recurred. The symptoms subsided on taking nitroglycerin, and she did not seek medical attention. We believe that the angina was caused by an interaction between diltiazem and methoxyphenamine capsules. The patients Naranjo score was 7.

### Case 2

A 65-year-old man with hyperthyroidism and no history of alcohol consumption or viral hepatitis was taking propylthiouracil for a year. He took CONTAC NT for an upper respiratory tract infection (one tablet per dose, taken three times a day). On the sixth day, he developed hallucinations, blurred vision, jaundice, nausea, and vomiting and was subsequently admitted to the hospital. The following were the findings on admission: normal respiration, blood pressure, and heart rate; altered consciousness; delayed reactions; no abnormalities on head computed tomography (CT) and magnetic resonance imaging (MRI); normal electrocardiogram and electroencephalogram; total bilirubin, 174.08 μmol/L; direct bilirubin, 100.76 μmol/L; indirect bilirubin, 73.32 μmol/L; alanine aminotransferase (ALT), 1266.39 U/L; aspartate aminotransferase (AST), 613.19 U/L; *γ*-glutamyl transpeptidase (GGT), 677.35 U/L; albumin, 39.46 g/L; prothrombin time (PT), 12.6 s; activated partial thromboplastin time (APTT), 42.4 s; international normalized ratio (INR), 1.16; thrombin time (TT), 16 s; fibrinogen, 1.87 g/L; total triiodothyronine (T_3_), 2.77 nmol/L; total thyroxine (T_4_), 181 nmol/L; free serum triiodothyronine (FT_3_), 2.6 pg./mL; free serum thyroxine (FT_4_), 14.2 pmol/L; and thyroid stimulating hormone (TSH), 1.31 μIU/mL. Blood creatinine, electrolytes, white blood cells, hemoglobin, and platelets were all within normal ranges. The patient and his family refused lumbar puncture and cerebrospinal fluid examination. He was initially diagnosed with drug-induced acute liver failure (paracetamol and propylthiouracil) and possible viral encephalitis. On the first day of admission, he was treated with acyclovir, polyene phosphatidylcholine, and ademetionine 1,4-butanedisulfonate, and propylthiouracil was discontinued. The patient experienced two episodes of hallucinations and agitation on the same day. On the second day, his consciousness returned to normal. On the third day, the physicians and clinical pharmacist considered that the patient’s clinical manifestations were not consistent with encephalitis, and the psychiatric abnormalities were caused by liver failure. Therefore, acyclovir was discontinued, and methimazole (10 mg once daily) was added. On the seventh day, the patient’s liver function significantly improved. The following results were observed: total bilirubin, 81.26 μmol/L; ALT, 399.71 U/L; AST, 212.18 U/L; GGT, 423.24 U/L; INR, 1.04; and fibrinogen, 2.12 g/L. On the 17th day, the patient’s skin color returned to normal, with no complaints of discomfort. The following results were observed: total bilirubin, 49.51 μmol/L; ALT, 166.98 U/L; AST, 91.94 U/L; and GGT, 317.57 U/L, with normal coagulation function. The patient was discharged on the 18th day. Two months after discharge, no further psychiatric abnormalities were observed. The patients Naranjo score was 6.

### Case 3

A 63-year-old man, without hypertension or diabetes, had Parkinson’s disease for 2 years and had been taking levodopa, entacapone, and selegiline for a long time. On November 21, 2023, he took CONTAC NT because of runny nose, cough, and muscle aches. Approximately 4 h later, the patient developed facial flushing, a temperature of 39.8°C, and altered mental status, and he was urgently taken to the hospital. On admission, an examination revealed the following: confusion; disorientation; agitation; temperature, 39.5°C; blood pressure, 198/133 mmHg; heart rate, 118/min; respiratory rate, 21 breaths/min; cough with sputum; normal findings on head MRI and CT; and chest CT suggesting pulmonary bullae. His laboratory results were as follows: white blood cell count, 12.98*10^9^/L; C-reactive protein, 19.5 mg/L; procalcitonin, 0.038 ng/mL; T_3_, 1.68 nmol/L; T_4_, 110 nmol/L; FT_3_, 3.61 pg./mL; FT_4_, 16 pmol/L; TSH, 2.56 μIU/mL; cerebrospinal fluid white blood cell count, 5/uL; glucose, 2.52 mmol/L; and protein, 0.27 g/L. He was diagnosed with fever of unknown origin and unspecified mental status abnormalities and was treated with ceftriaxone, esmolol, and benidipine. The blood pressure, heart rate, temperature, and mental status gradually improved on the second day and returned to normal on the third day. Serotonin syndrome due to drug–drug interactions was considered after inquiring about the patient’s medication history. The patients Naranjo score was 6.

## Discussion

Compound cold medicines can effectively alleviate discomfort caused by upper respiratory tract infections and are generally considered safe. These drugs are widely used without the need for prescriptions. However, in elderly patients on long-term medications, the indiscriminate use of compound cold medicines may increase the risk of drug interactions. To the best of our knowledge, this is the first report to describe the risks associated with the use of compound cold medicines by elderly individuals. Additionally, we identified for the first time that the combination of methoxyphenamine and diltiazem may lead to angina and that the combination of propylthiouracil and paracetamol may result in liver failure and psychiatric abnormalities.

In Case 1, the patient had no history of coronary artery disease or angina, and had a normal lipid profile, blood pressure, blood glucose, and liver and kidney function. However, after concurrently taking diltiazem and compound methoxyphenamine, the patient developed angina. Angina recurred the following day on taking both medications again. The patient’s rechallenge test results were positive, indicating a definitive adverse reaction. According to the manufacturer’s instructions, an overdose of aminophylline can lead to tachycardia and arrhythmia (2), while diltiazem can increase aminophylline levels (3). There have been no reports of the use of diltiazem combined with aminophylline leading to angina. Moreover, no evidence of drug–drug interactions among methoxyphenamine hydrochloride, phenylephrine, chlorpheniramine maleate, and diltiazem has been documented. To the best of our knowledge, this is the first report of the interaction of diltiazem with aminophylline inducing angina.

In Case 2, the patient was taking propylthiouracil for a year without any adverse reactions. However, after taking “CONTAC NT,” the patient experienced mental abnormalities and acute liver failure. Both propylthiouracil and acetaminophen are predominantly metabolized in the liver, and can cause acute liver failure (4–7). Current evidence indicates that propylthiouracil is not metabolized by CYP450 enzymes, while only a small amount of acetaminophen is metabolized by CYP2E1 (5). Therefore, pharmacokinetic interactions between these two drugs are unlikely. Further, there is no evidence to suggest that propylthiouracil and acetaminophen compete for transport proteins. No evidence for drug–drug interaction between these two drugs were identified in the UpToDate, Lexicomp, or any other relevant drug databases (8–10). Therefore, we speculate that the combination of propylthiouracil and acetaminophen exacerbates the burden on the liver, resulting in acute liver failure. Dextromethorphan and chlorpheniramine maleate commonly cause drowsiness. Propylthiouracil and acetaminophen are unlikely to cause mental status abnormalities. Pseudoephedrine can stimulate the central nervous system; therefore, we suspected that it caused mental status abnormalities in the patient. Additionally, acute liver failure may have worsened the patient’s mental status abnormalities.

Serotonin syndrome is a syndrome or drug toxidrome induced by serotonergic drugs, such as selective serotonin reuptake inhibitors (SSRIs) and monoamine oxidase inhibitors (MAOIs) (11). Serotonin syndrome is clinically diagnosed based on a physical examination and medication history assessment. Typical clinical manifestations include autonomic overexcitation (hyperpyrexia, tachycardia, and hypertension), neuromuscular abnormalities (myoclonus and tremors), and altered mental status (confusion, agitation, coma, and anxiety) (12–14). In Case 3, the patient did not have hypertension or diabetes, and his kidney function was normal. He had been taking levodopa, entacapone, and selegiline for a long time, without any apparent adverse reactions. However, after adding CONTAC NT, symptoms such as altered mental status, elevated blood pressure, increased heart rate, and high fever appeared, consistent with the clinical presentation of serotonin syndrome. CONTAC NT contains dextromethorphan. The combination of dextromethorphan with selegiline and of pseudoephedrine with selegiline can lead to serotonin syndrome (15–18). Therefore, we believe that the severe adverse reaction in this patient was caused by the combination of selegiline and the compound cold medicine.

This study has several limitations, such as a small sample size, recall bias, and patient heterogeneity. However, this report highlights the risks of the self-administration of compound cold medicines by elderly patients; this requires careful attention from both patients and caregivers.

## Conclusion

In conclusion, elderly patients should consult a pharmacist or physician before using compound cold medicines to avoid the risk of adverse reactions caused by drug–drug interactions.

## Data Availability

The original contributions presented in the study are included in the article/supplementary material, further inquiries can be directed to the corresponding author.
